# Genome–Environment Associations, an Innovative Tool for Studying Heritable Evolutionary Adaptation in Orphan Crops and Wild Relatives

**DOI:** 10.3389/fgene.2022.910386

**Published:** 2022-08-05

**Authors:** Andrés J. Cortés, Felipe López-Hernández, Matthew W. Blair

**Affiliations:** ^1^ Corporacion Colombiana de Investigacion Agropecuaria AGROSAVIA, C.I. La Selva, Rionegro, Colombia; ^2^ Department of Agricultural & Environmental Sciences, Tennessee State University, Nashville, TN, United States

**Keywords:** gene banks, germplasm collections, landraces, genome–environment associations (GEA), genome-wide environmental scans, genome-wide selection scans (GWSS), genomic prediction (GP), abiotic stress

## Abstract

Leveraging innovative tools to speed up prebreeding and discovery of genotypic sources of adaptation from landraces, crop wild relatives, and orphan crops is a key prerequisite to accelerate genetic gain of abiotic stress tolerance in annual crops such as legumes and cereals, many of which are still orphan species despite advances in major row crops. Here, we review a novel, interdisciplinary approach to combine ecological climate data with evolutionary genomics under the paradigm of a new field of study: genome–environment associations (GEAs). We first exemplify how GEA utilizes *in situ* georeferencing from genotypically characterized, gene bank accessions to pinpoint genomic signatures of natural selection. We later discuss the necessity to update the current GEA models to predict both regional- and local- or micro-habitat–based adaptation with mechanistic ecophysiological climate indices and cutting-edge GWAS-type genetic association models. Furthermore, to account for polygenic evolutionary adaptation, we encourage the community to start gathering genomic estimated adaptive values (GEAVs) for genomic prediction (GP) and multi-dimensional machine learning (ML) models. The latter two should ideally be weighted by *de novo* GWAS-based GEA estimates and optimized for a scalable marker subset. We end the review by envisioning avenues to make adaptation inferences more robust through the merging of high-resolution data sources, such as environmental remote sensing and summary statistics of the genomic site frequency spectrum, with the epigenetic molecular functionality responsible for plastic inheritance in the wild. Ultimately, we believe that coupling evolutionary adaptive predictions with innovations in ecological genomics such as GEA will help capture hidden genetic adaptations to abiotic stresses based on crop germplasm resources to assist responses to climate change.

“I shall endeavor to find out how nature’s forces act upon one another, and in what manner the geographic environment exerts its influence on animals and plants. In short, I must find out about the harmony in nature” Alexander von Humboldt—*Letter to Karl Freiesleben, June 1799*.

## Introduction—Lack of Crop Genotypes Adapted to Severe Climates

Crop wild relatives (CWR) and landraces are well known for providing new alleles for plant breeding (Tanksley and McCouch, 1997). They also can improve dietary proteins and essential micronutrients for undernourished communities (Blair, 2013). However, their diversity is often unexplored and underutilized (Bronnvik and von Wettberg, 2019; Ramirez-Villegas et al., 2022). Major cultivars typically lack adaptation to abiotic stresses (i.e., heat and drought), jeopardizing worldwide yield stability, given increasing effects of climate change (Cortés et al., 2020a). Luckily, landraces and CWR, as well as orphan crop species, offer novel adaptive alleles (Herron et al., 2020).

For instance, among legumes, bambara groundnut (*Vigna subterranea*), chickpea (*Cicer arietinum*), cowpea (*V. unguiculata*), grass pea (*Lathyrus sativus*), groundnut (*Arachis hypogaea*), marama bean (*Tylosema esculentum*) (Cullis et al., 2018), tarwi (*Lupinus mutabilis*) (Atchison et al., 2016; Gulisano et al., 2019), and tepary bean—(*Phaseolus acutifolius*) (Buitrago-Bitar et al., 2021; Burbano-Erazo et al., 2021) constitute genepools of unexplored adaptive diversity for abiotic stresses*.* Among cereals, orphan crops include teff (*Eragrostis tef*), sorghum (*Sorghum bicolor*), and finger millet (*Eleusine coracana*) or pearl and proso millets (*Panicum* spp, *Pennisetum glaucum*). Many of these crops have interesting drought-tolerance traits and some capacity to grow in compacted soils. Cowpeas, groundnuts, and lesser known cereals are already traditional food sources and biocultural components for vulnerable areas, especially in Sub-Saharan Africa and parts of Asia and Latin America (Xiong et al., 2016).

Yet, despite their tolerance to drought and heat plus their high nutritional quality, the utilization of these orphan crops is limited partly because of the poor characterization of their genetic background (Wu et al., 2022). Therefore, a key research area in orphan crop improvement is to expand the use of modern molecular prebreeding tools for them (Ahmad et al., 2020), that is, genome resequencing (Fuentes-Pardo and Ruzzante, 2017; Wu et al., 2020) and genomic prediction (Desta and Ortiz, 2014), to select genotypes for production in dry climates.

Still, a major question to harness crop prebreeding for climate change pressures is whether there is enough heritable variation in traits associated with tolerance to abiotic stress. In this context, genomic characterizations of reference collections comprising CWR, landraces, and orphan crops that span contrasting habitats offer a straightforward scenario to identify natural standing adaptation to abiotic pressures (Ramirez-Villegas et al., 2022).

The spirit behind this novel approach is to detect genomic regions that correlate with habitat heterogeneity as an indication of the natural selection imprint to environmental gradients (Forester et al., 2016). Since these signatures rely on a natural equilibrium between genotypes and their environment (Hancock et al., 2011), the ideal base population must prioritize natural genotypes and landraces and avoid improved cultivars, for which it is not realistic to assume that enough generations have passed as to display divergent selection to environmental heterogeneity. Hence, the goal of the present work is to review key developments to explore and utilize natural adaptation in wild genepools for climate change adaptation.

## *Modus Operandi* to Genomically Assess Natural Evolutionary Adaptation

Coupling ecological genomics innovations (Cortinovis et al., 2020b) with evolutionary adaptive trajectories (Ramírez et al., 2010; López-Hernández and Cortés, 2019; Cortinovis et al., 2020a; Ramirez-Villegas et al., 2020) helps capturing adaptations in CWR and landraces, as shown for teosinte (Pyhäjärvi et al., 2013), rice (Meyer et al., 2016), *Glycine* (Anderson et al., 2016), and barley (Russell et al., 2016).

The current assortment of genomic pipelines that analyze environmental variation in order to infer the genetic basis of adaptation to natural selection include genome-wide selection scans—GWSS (Zahn and Purnell, 2016), and genome-wide environmental scans—GWES (Rellstab et al., 2015) also known as genome–environment associations—GEAs, a term we prefer based on its simplicity. While GWSS relies on the outlier Bayesian tests contrasted against a genomic background distribution (Antao et al., 2008), GEA uses mixed linear models (MLMs) that incorporate random effects, such as kinship and population stratification (Kruglyak, 2008). GEA behaves like traditional genome-wide association study (GWAS), but instead of modeling a set of phenotypic traits, it considers an environmentally derived variable into its additive genetic factors. These inferences could be misleading (Maher, 2008; Pennisi, 2014) if they overlook the confounding factors (Lambert and Black, 2012; Wolf and Ellegren, 2017) such as demographic (Barton et al., 2019) and genomic (Wray et al., 2013; Huber et al., 2016; Ellegren and Wolf, 2017) constraints also prevalent in GWAS studies. Hence, MLM-based models, which are capable of handling these spurious sources of error, are currently the optimum approach for the use of GEA and environmental variables to unveil the extent and genetic bases of local adaptation in diverse natural populations (Abebe et al., 2015).

In the last decade, the GEA pipeline has been utilized to characterize signatures of environmental adaptation in a rich spectrum of plant species (Table 1). For instance, Eckert et al. (2010) studied environmental associations with aridity across the range of the pine tree *Pinus taeda*, showing utility of the GEA approach for long-lifecycle, forestry species. This approach proved useful for additional tree species in studies by Holliday et al. (2016) and Pluess et al. (2016), respectively, who demonstrated local adaptation to climate gradients in *Populus trichocarpa* and *Fagus sylvatica*. More recently Ingvarsson and Bernhardsson (2020) address climate adaptation in *P. tremula* under present and future scenarios. With the increasing need for biomass as a fuel source, GEA is likely to continue its important role in the genetic analysis of other trees and woody species.

**TABLE 1:** Examples of GEA studies carried out in plant species. This compilation of previous studies explicitly refers to genome–environment association studies (GEA) using the Scopus database https://www.scopus.com/ with the following search parameters: TITLE-ABS-KEY (“Genome – Environment Associations”) AND (LIMIT-TO (DOCTYPE, “ar”)). The table is sorted chronologically. Method abbreviations are shown at the bottom of the table.

Speciesapproach	Sampling data	Genotypic data	Targeted stress	Environmental data	Analytical	Main finding	References
GLM	Tolerance to aridity	*Pinus taeda*	3,059 SNPs and 23 SSRs	Aridity index and Thornthwaite index using biovariables http://worldclim.org/version2	622 trees	Environmental and genetic data for the identification of functionally important genetic variation within natural populations	Eckert *e*t al. (2010)
Genome-wide scans	Local adaptation to climate gradients	*Arabidopsis*	∼215,000 SNPs	Aridity, temperature, precipitation, radiation, and day length http://www.sciencemag.org/content/suppl/2011/10/05/334.6052.83.DC1.html	948 accessions	Natural adaptive genetic variation in *Arabidopsis* at a continental scale	Hancock et al. (2011)
Redundancy analysis (RDA)	Local adaptation to climate gradients	*Arabidopsis*	214,051 SNPs	Potential evapotranspiration using annual precipitation and a measure of aridity http://worldclim.org/version2, variability in precipitation http://esrl.noaa.gov/psd/, and photosynthetically active radiation http://eosweb.larc.nasa.gov/PRODOCS/srb/table_srb.html	1,003 accessions	The climatic structure of SNP correlations is due to changes in coding sequence that may underlie local adaptation	Lasky et al. (2012)
MLM	Drought and heat stress	*Medicago truncatula* †	1,918,637 SNPs	Biovariables http://worldclim.org/current	202 accessions	Genetic basis of adaptation to drought and heat stress disclosed in *M. truncatula*	Yoder et al. (2014)
EMMA	Tolerance to aluminum toxicity and drought stress	*Sorghum bicolor*	404,627 SNPs	Potential evapotranspiration using annual precipitation and a measure of aridity http://worldclim.org/version2, variability in precipitation http://esrl.noaa.gov/psd/, photosynthetically active radiation https://eosweb.larc.nasa.gov/project/srb/srb_table, and edaphic data http://daac.ornl.gov/SOILS/guides/DunneSoil.html, http://www.fao.org/nr/water/docs/harm-world-soil-dbv7cv.Pdf	1,943 accessions	Genomic signatures of environmental adaptation may be useful for crop improvement, enhancing germplasm identification, and marker-assisted selection	Lasky et al. (2015)
BayeScan	Local adaptation to climate gradients	*Populus trichocarpa*	∼170,000 SNPs	Variables from Holliday *et al.* (2016)	391 trees	Physical proximity of genes in coadapted complexes may buffer against the movement of maladapted alleles from geographically proximal but climatically distinct populations	Holliday et al. (2016)
LFMMs, GLM	Local adaptation to climate gradients	*Fagus sylvatica*	144 SNPs—12 SSRs	Environmental index from raw variables in Pluess *et al.* (2016) and references herein	79 natural populations	Local adaptation to climate gradients	Pluess et al. (2016)
Bayenv, Bayescan	Local adaptation to climate gradients	*Cenchrus americanus*	87,218 SNPs	Variables from Berthouly-Salazar *et al.* (2016)	762 trees	Outlier loci putatively under selection detected in populations at the extremity of climatic gradients, and tested *ad hoc* in populations along the gradients	Berthouly-Salazar et al. (2016)
MLM, GLM	Drought stress	*Phaseolus vulgaris* †	22,845 SNPs	Drought index using Thornthwaite model and annual precipitation http://worldclim.org/version2	86 accessions	Genomic signatures of adaptation are useful for germplasm characterization, potentially enhancing future marker-assisted selection, and crop improvement	Cortés and Blair (2018a)
BayPass	Abiotic stresses	*Arabidopsis*	1,638,649 SNPs	Mean annual temperature; mean coldest month temperature; and precipitations in winter, spring, summer, and autumn https://sites.ualberta.ca/∼ahamann/data/climateeu.html	168 natural populations	The identification of climate-adaptive genetic loci at a micro-geographic scale also highlights the importance to include within-species genetic diversity in ecological niche models for projecting potential species distributional shifts	Frachon et al. (2018)
LFMMs and GLM	Drought stress	*Beta vulgaris* subsp. *vulgaris*	14,409 SNPs	Aridity index using biovariables http://worldclim.org/version2	1,249 accessions	Wild individuals have higher ability to resist stress-aridity conditions and could be used to improve the resistance of cultivated varieties	Manel et al. (2018)
LFMMs and MSOD-MSR	Drought stress	*Medicago truncatula* †	43,515 SNPs	Biovariables http://worldclim.org/version2, atmospheric nitrogen deposition https://daac.ornl.gov/, and soil variables https://library.wur.nl/WebQuery/wurpubs/510208	202 accessions	The importance of soil in driving adaptation in the system and elucidate the basis of evolutionary potential of *M. truncatula* to respond to global climate change and anthropogenic disruption of the nitrogen cycle	Guerrero et al. (2018)
GLM	Abiotic stress	*Zea mays*	355,442 SNPs	*tmax*, *tmin*, *tavg*, *srad*, *vapr*, *ph5*, and *prec* https://soilgrids.org http://worldclim.org/version2	1,143 accessions	Combining large-scale genomic and ecological data in this diverse maize panel, this study supports a polygenic adaptation model of maize and offers a framework to enhance the understanding of maize adaptation	Li et al. (2019)
SUPERFarmCPU, BLINK, GLM, and MLM	Heat stress	*Phaseolus vulgaris* †	23,373 SNPs	PCA from temperature biovariables, modified heat Thornthwaite index, and heat index http://worldclim.org/version2	78 accessions	It is feasible to identify genome-wide environmental associations with modest sample sizes by using a combination of various carefully chosen environmental indices and last-generation GWAS algorithms	López-Hernández and Cortés (2019)
Bayenv2	Drought and heat stress	*Betula nana*	14,889 SNPs	Biovariables http://worldclim.org/version2	130 accessions	Significant correlation between the number of loci associated with each environmental variable in the GEA, and the importance of each variable in environmental niche modeling	Borrell et al. (2019)
LFMMs	Drought and heat stress	*Quercus aquifolioides*	381 SNPs	Isothermality, mean temperature of the driest quarter, precipitation during the dry season, and precipitation during the wet season http://worldclim.org/version2	60 accessions	Genetic variation in *Q. aquifolioides* showed contrasted patterns of local adaptation in the two lineages	Du *et al.* (2020)
CANCOR	Drought stress	*Lolium perenne*	189,968 SNPs	Environment index http://etccdi.pacificclimate.org/index.shtml, soil data https://esdac.jrc.ec.europa.eu/, and biovariables http://worldclim.org/version2	469 natural populations	CANCOR retrieved 633 outlier loci associated with two climatic gradients, characterized by cold–dry vs. mild–wet winter, and long rainy season vs. long summer, pointing out traits putatively conferring adaptation at the extremes of these gradients	Blanco-Pastor *et al.* (2020)
BLINK	Drought and heat stress	*Sorghum bicolor L.*	72,190 SNPs	Altitude, annual temperature, and precipitation of accessions’ passport data from Girma *et al.* (2020)	1,425 accessions	Candidate loci identified with the GEA will have potential utilization for germplasm identification and sorghum breeding for stress	Girma *et al.* (2020)
BAYESCENV	Drought and heat stress	*Circaeaster agrestis*	6,120 SNPs	Isothermality, evapotranspiration, temperature seasonality, temperature annual range, annual precipitation, and seasonality precipitation www.chelsa-climate.org	139 accessions	Genome-wide data provide new insights into the important role of environmental heterogeneity in accessing the footprints of local adaptation in an ancient relictual species	Zhang *et al.* (2020)
LFMMs	Abiotic stress	*Populus tremula*	8,007,303 SNPs	Abiotic index ENVIREM http://envirem.github.io, climatic variables based on the CCSM4.0 model http://www.cesm.ucar.edu/models/ccsm4.0/ccsm/, and biovariables http://worldclim.org/current	94 trees	Climate adaptation in *P. tremula* under present and future scenarios	Ingvarsson and Bernhardsson (2020)
BLINK	Drought and heat stress	*Sorghum bicolor L.*	54,080 SNPs	Altitude, annual temperature, and precipitation http://worldclim.org/version2	940 accessions	The current study aimed to better understand the GEA of a large collection of Ethiopian sorghum landraces, characterized with genome-wide SNP markers, to investigate key traits related to adaptation	Menamo et al. (2021)
FarmCPU	Cold stress	*Broussonetia papyrifera*	2,936,477 SNPs	Frost-free period and other climatic information http://data.cma.cn	134 accessions	Significant selective regions and candidate genes were identified, and the potential molecular mechanism of local adaptation to low temperature in woody plants was discussed	Hu et al. (2021)
MLM	Drought stress	*Phaseolus acutifolius* A. Gray †	Genes *Asr2*, *Dreb2B*, *ERECTA*	Drought index using Thornthwaite model and annual precipitation http://worldclim.org/version2	52 accessions	The results suggested that tepary bean, specially wild accessions, could be sources of novel alleles for drought tolerance	Buitrago-Bitar et al. (2021)
MLM	Abiotic stress	*Phaseolus vulgaris* L. †	28,823 SNPs	Biovariables from http://worldclim.org/version2	110 accessions	SNP markers and candidate genes associated with bio-climatic variables should be validated in segregating populations for water MAS	Elias *et al.* (2021)
LFMMs	Abiotic stress	*Medicago truncatula* †	14,160 SNPs	Worldclim.org (WC), The Climatic Research Unit (University of East Anglia) (CRU), The Satellite Application Facility on Climate Monitoring, and The NASA Distributed Active Archive Centre for Biogeochemical Dynamics (DAAC)	675 accessions	Authors identified a set of candidate genes for adaptation associated with environmental gradients along the distribution range	Blanco-Pastor *et al.* (2021)
LFMMs	Abiotic stress	*Medicago sativa* †	10,478 SNPs		202 accessions		

FarmCPU, fixed and random model circulating probability unification; BLINK, Bayesian-information and linkage-disequilibrium iteratively nested keyway; LFMMs, latent factor mixed models; CANCOR, canonical correlation analysis; SUPER, settlement of MLM under progressively exclusive relationship; MSOD-MSR, Moran spectral outlier detection/randomization; EMMA, mixed linear association model; MAS, marker-assisted selection.

Symbol † indicates studies in legume species.

Meanwhile, for model plants, GEA has also gained in popularity and has a somewhat longer history: Hancock et al. (2011) and Lasky et al. (2012) have explored natural adaptive genetic variation in *Arabidopsis* at a continental scale and for water use efficiency, Yoder et al. (2014) disclosed the genetic basis of adaptation to drought and heat stress in *Medicago truncatula*.

**FIGURE 1:**
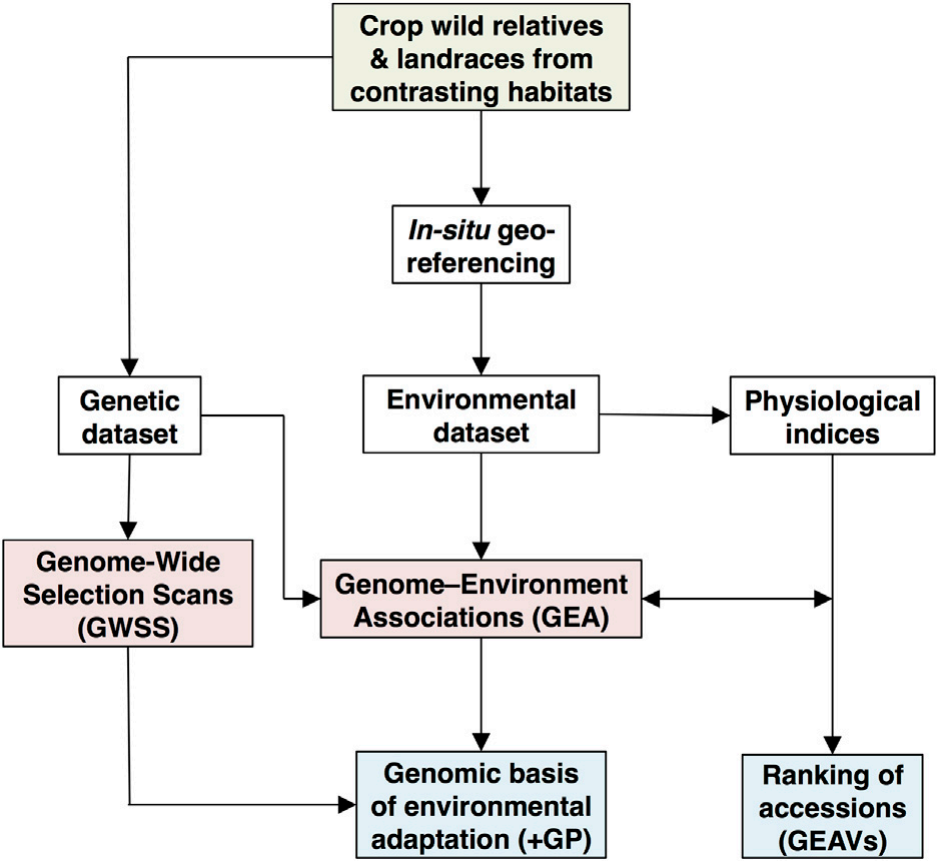
Analytical pipeline to infer genome-wide signatures of environmental adaptation in crop wild relatives (CWR) and landraces that span heterogeneous environments. The green shaded box refers to gene bank collections, while white, red and blue shaded boxes represent input data, analytical models and output inferences, respectively (Cortés et al., 2020a; Cortés et al., 2020b; Cortés and López-Hernández, 2021). Genomic prediction (GP) and genomic-estimated adaptation values (GEAVs) promise speeding up plant breeding goals.

For orphan crops, rather than nondomesticated natural plant species, fewer GEA studies have been undertaken. Although still limited to the easier-to-grow annual grain species compared to perennial and root/tuber crops, GEA is starting to make important contributions to genetic analysis of landraces and WCR germplasm, often richly represented in the world’s major gene banks for crop species. The analytical pipeline for GEA studies is shown in Figure 1, with the input data, analytical models, and outputs found when inferring genome-wide signatures of environmental adaptation in crop wild relatives (CWR) and landraces that span heterogeneous environments. The reader is referred to pertinent examples of GEA in crop species or their CWR which include Lasky *et al.* (2015), who prospected natural tolerance to aluminum toxicity and drought in cultivated sorghum; Berthouly-Salazar *et al.* (2016), who captured genomic regions involved in adaptation on two climate gradients in pearl millet; Cortés and Blair (2018a), who evaluated drought-tolerance sources of common bean (*Phaseolus vulgaris*) WCR; or López-Hernández and Cortés (2019), who identified pervasive divergent adaptation to continental-level heat gradients in wild accessions of this same species.

Historically, based on the technological improvements in sequencing and SNP detection and as a means to improve GEA, the field has moved from the candidate gene approach (Cortés et al., 2012a; Cortés et al., 2012b; Blair et al., 2016; Buitrago-Bitar et al., 2021) into full genomic scans (Cortés and Blair, 2018a; López-Hernández and Cortés, 2022), which better account for linkage disequilibrium (LD) heterogeneity. In this movement, the targeting of discrete responses to abiotic pressures has aided GEA studies, by explicitly relying on the mechanistic ecophysiological models and traits (Cortés et al., 2013) for overall conditions such as drought (Cortés and Blair, 2018a) and heat stress (López-Hernández and Cortés, 2022).

## More Powerful Next-Generation GEA Models Meet Evolutionary Ecology

As discussed previously, GEA is becoming a key tool to prospect for new genes among crop wild accessions and landraces as an alternative to traditional phenotyping and GWAS analyses (Cortés et al., 2022). However, there is still room for innovation. For example, we envision dual GEA models that combine inferences at various spatial and temporal scales, following Cortés et al. (2020a), by 1) predicting regional- and microhabitat-wise evolutionary adaptation with *in situ* ecological georeferencing of accessions, and by 2) revealing the genomic architecture of adaptation *via* cutting-edge predictive models (Rellstab et al., 2015; Forester et al., 2016).

Concerning the first point, standardized climate data for GEA studies are as important as high-quality genotyping to guarantee analytical power (Waldvogel et al., 2020). Climate-based inferences may target extreme regions, like those where drought is coupled with extreme temperatures (Lei et al., 2019). The high-resolution climate data can be gathered from worldwide repositories (such as WorldClim, https://www.worldclim.org/) using georeferencing and statistical downscaling (Zellweger et al., 2019) in order to build explicit heat–stress physiological indices (Cortés et al., 2013; López-Hernández and Cortés, 2019) at regional and local scales, respectively. The use of explicit indices, instead of raw environmental variables, helps describing physiological processes more accurately, especially those that confer tolerance to abiotic stress. For instance, the same environmental dataset can be inputted into an evapotranspiration model to infer drought stress (Cortés et al., 2013) and its genetic bases (Cortés et al., 2012a; Cortés et al., 2012b; Blair et al., 2016; Cortés and Blair, 2018a), or to assess heat tolerance (López-Hernández and Cortés, 2019). It is equally paramount to collect the spatial high-resolution climate data to improve predictions not only at regional scales (Pluess et al., 2016), but also at microhabitat levels (Cortés et al., 2015; Frachon et al., 2018), where adaptive variation to cope with abiotic pressures is overlooked but is sufficient (Cortés and Wheeler, 2018). Remote sensing (Zellweger et al., 2019) also promises better capture of environmental heterogeneity (Ratcliffe et al., 2019).

Concerning the second opportunity for improvement, characterizing genome-wide signatures of environmental adaptation to habitat-inferred stress in CWR and landraces that span heterogeneous climates will benefit from inputting habitat-based abiotic stress indices into the last-generation mixed linear models (MLMs) and machine learning (ML) (Schrider and Kern, 2018; Cortés et al., 2020b), capable of handling spurious effects (Barton et al., 2019) in multidimensional data, while detecting predictive genomic regions that correlate with habitat/environmental heterogeneity, as an indication of the genomic imprint by climate gradients (Hancock et al., 2011).

## Overcoming Polygenic Adaptation

Overall, CWR and landraces undeniably harbor unique adaptations to abiotic stresses, rarely present in the cultivated and improved genepools (Tester and Langridge, 2010). However, unlocking and utilizing this potential (Tanksley and McCouch, 1997; Langridge and Robbie, 2019) has remained challenging partly due to phenotyping bottlenecks in the wild, and the complex inheritance (Morran et al., 2011) of trait variation for abiotic stress tolerance, typically involving many loci with low effects (López-Hernández and Cortés, 2019). To bridge this gap, we propose extending genomic prediction (GP) models and genomic estimated breeding values (GEBVs) to account for the habitat-based dimensions by coining the analogous parameter genomic estimated adaptation values—GEAVs (Capblancq et al., 2020; Arenas et al., 2021), equivalent to the polygenic risk score (PRS) within a preimplantation genetic diagnosis framework. To compute GEAVs, the GP models used must be calibrated (trained and tested) to predict the environmental indices and polygenic adaptability (liability) thresholds.

GP works either on the basis of shared relatedness (typically measured as relationships due to recent coancestry) or on the basis of linkage disequilibrium (LD) between the SNP marker loci and the genetic variants that underlie phenotypic variation (Thistlethwaite et al., 2020). The relationships between the training and testing datasets are critically important and therefore must be optimized as part of any GP effort. As a general suggestion, the more diverse the training dataset is, the more robust the prediction will be. Hence, coupling cross-validation calibration curves under various training/testing ratios with simple measures of diversity, such as heterozygosity and runs of homozygosity (ROH), is appropriate. However, in the absence of demonstrated relationships or LD, there is no apparent basis for GP’s predictive accuracy, raising the possibility of statistical artifacts. To overcome this issue, pairwise LD among SNP markers helps to interpret GWAS-type GEA associations (Morton, 2005), and this a prerequisite for any GP initiative.

The predictive ability of GP models may be further biased depending on whether the entire SNP set is used, or the most predictive SNP data are chosen after the GWAS-type analysis (Wray et al., 2013), the latter being a common practice in modern GP studies. The polygenic infinitesimal model that makes GP so unique may be jeopardized by subsampling the SNP markers because relying only on GEA-derived SNP markers would disregard SNPs with low effects that are usually missed, but that may still account for the overall missing heritability.

Better approaches to be implemented and reported include 1) weighted GP models using *de novo* GWAS-based GEA estimates gathered from other (or even the same) panels (Spindel et al., 2016) and 2) optimization of the marker set by computing the saturating curves of the predictive ability, given various sets of markers ranked by their beta effects from the exact same GP model and not from any parallel GWAS-derived GEA models (Resende et al., 2012; Tan et al., 2017). GP can also be improved in efficiency by validating the GEAV scores across diverse populations, allowing better G × E predictions within the nascent field of enviromics (Resende et al., 2021; Cooper and Messina, 2021; Costa-Neto et al., 2021). GEAVs can boost incorporation of landraces and CWR as parents in marker-assisted backcrossing (MAB). Marker set optimization for environmental GP (Jarquín et al., 2013; Lopez-Cruz et al., 2015) would benefit crop prebreeding initiatives for abiotic tolerance MAB, mergeable with the speed-breeding strategies (Migicovsky and Myles, 2017; Watson et al., 2018), high-throughput screening (Cuppen, 2007), and ML-updated best linear unbiased prediction (BLUP) models (Wenlong et al., 2018; Crossa et al., 2019; Abdollahi-Arpanahi et al., 2020; Cortés et al., 2020b; Wang et al., 2020; Zingaretti et al., 2020; Montesinos-López et al., 2021).

## Perspectives to Better Harness Genome Functionality in the Wild

Next-generation GEA models and GEAVs will allow the use of “exotic” parents for targeted predictive prebreeding in crop species. They offer feasible methodologies to trace the sources of abiotic stress adaptation and tolerance targeting crop resources in low-income countries, which are also the most vulnerable to climate change. Here, we have discussed strategies to implement the latest-generation predictive (e.g., Cortés et al., 2013) and genomic (Cortés and Blair, 2018a; López-Hernández and Cortés, 2019) approaches to study adaptation in CWR and landraces (Cortés and Blair, 2018b; Cortés et al., 2020a), but similar work could be done for any orphan crop.

Modern GEA approaches will allow further studies of evolutionary conservatism, parallelism, and convergence in the genetic architecture of adaptation to various types of abiotic stresses across a wide range of environments, landraces, and wild accessions (Cortés and López-Hernández, 2021). Approaches such as these are needed to discern among drivers (Ellegren and Galtier, 2016) of the adaptive landscape of genomic divergence (Feder et al., 2012; Gompert et al., 2014; Cortés et al., 2018b), such as ecological diversity, population structure, ancestral polymorphisms, mutation/recombination rates (Feder et al., 2012; Ellegren and Wolf, 2017; Ravinet et al., 2017; Cortés and Blair, 2018b), and nested levels of divergence (Nosil and Feder, 2011; Wolf and Ellegren, 2017; Cortés et al., 2018a).

How genetic diversity and genomic divergence arise and are shaped based on ecological pressures is one of the main questions in molecular evolution (Tiffin and Ross-Ibarra, 2014) and has been implicit even in the pregenetics-era studies of ecological transects by famous botanists such as von Humboldt. GEA studies can contribute valuable insights into the field of molecular evolution due to their ability to detect convergent or nonconvergent adaptations (Schmutz et al., 2014). Ecologically associated SNPs are likely to exhibit hitchhiking effects (Feder and Nosil, 2010) due to the low recombination rate and extensive LD (Kelleher et al., 2012; Blair et al., 2018).

**FIGURE 2:**
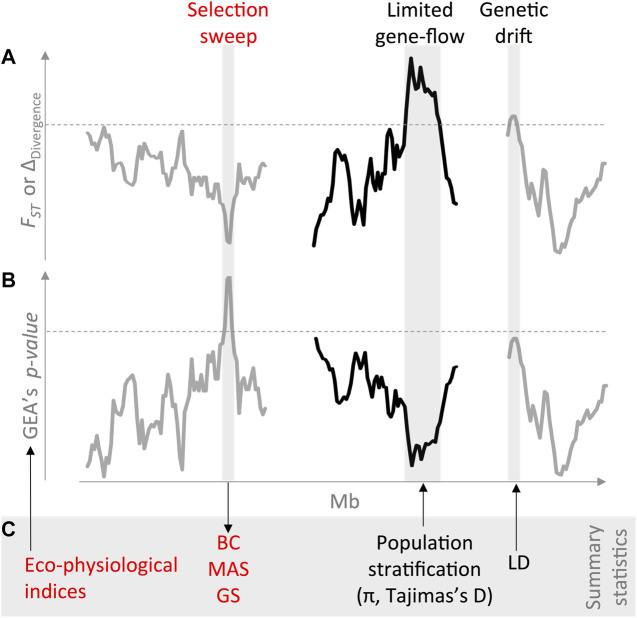
An integrated case study inspired in common bean (*Phaseolus vulgaris* L.) accessions exemplifies how to identify and harness natural signatures of environmental adaptation across diverse genepools. First, **(A)** genome-wide patterns of genetic divergence, as measured by the *F*_*ST*_ and delta divergence (Roesti et al., 2014) statistics, inform underlying confounding demographic processes across wild accessions (Blair et al., 2012) and landraces (Blair et al., 2009). Even though highly polymorphic markers have traditionally been preferred for demographic inferences (Blair et al., 2009; Kwak and Gepts, 2009), modern SNP genotyping technologies also enable reconstructing the genomic landscape of divergence at a higher resolution (Cortés et al., 2011; Wu et al., 2020). Once potential confounding demographic patterns have been accounted for, **(B)** it is then feasible to disentangle genuine (in red) signatures of environmental adaptation (Cortés et al., 2018b) from spurious concurring genetic drift due to genomic constraining features such as low recombining regions, reduced effective population size, and translocations (Blair et al., 2018). In order to improve genome–environment associations (GEA), the field has moved from the candidate gene approach (Cortés et al., 2012a; Cortés et al., 2012b; Blair et al., 2016; Buitrago-Bitar et al., 2021) into full genomic scans (Cortés and Blair, 2018a; López-Hernández and Cortés, 2022), which better account for linkage disequilibrium (LD) heterogeneity. It is also advisable to target discrete abiotic pressures by explicitly relying on the mechanistic ecophysiological models (Cortés et al., 2013) such as drought (Cortés and Blair, 2018a) and heat stress (López-Hernández and Cortés, 2022). Finally, **(C)** these combined summary statistics (i.e., ecophysiological indices, population stratification, and LD) can ultimately redound in prebreeding efforts aiming to introgress exotic adaptive variation into elite lines, for instance, *via* backcrossing (BC) schemes for abiotic (Muñoz et al., 2003; Blair et al., 2006; Blair and Izquierdo, 2012; Burbano-Erazo et al., 2021) and biotic (Garzon et al., 2008) stresses, all guided with indirect (Miklas et al., 2006) genomic selection tools such as marker-assisted selection (MAS) and genomic selection, GS (Cortés et al., 2020a; Cortés et al., 2020b; Cortés and López-Hernández, 2021) within a moder enviromics approach (Cooper et al., 2021). Different line colors stand for hypothetical distinct chromosomes. Dashed horizontal lines mark significance thresholds.

Therefore, GEA efforts can be enhanced by exploring SNP density and statistics of site frequency spectra (e.g., nucleotide diversity and Tajima’s D) in associated vs. nonassociated regions (Cortés and Blair, 2018a). Figure 2 shows an example of these principles used in common bean to identify and harness natural signatures of environmental adaptation across diverse genepools, with the genome-wide patterns of genetic divergence, as measured by the F_ST_ statistic (A). Once the potential confounding demographic patterns have been accounted for, it is then feasible to disentangle genuine signatures of environmental adaptation (B) from spurious concurring genetic drift due to genomic constraining features. Finally, these combined summary statistics (i.e., ecophysiological indices, population stratification, and LD) can ultimately redound in prebreeding efforts aiming to introgress exotic adaptive variation into elite lines (C), for instance, *via* backcrossing (BC) schemes for abiotic and biotic stresses.

All drivers must be considered as *ad hoc* multiple hypotheses (Chamberlin, 1897) since extensive LD and hitchhiking may not only be due to physical linkage and low recombination/effective population size (Slatkin, 2008), but also to population stratification, sample’s co-ancestry (Price et al., 2010; Blair et al., 2012), within-pathway gene-gene and G × E interactions (Ortiz et al., 2022), and context-dependent effects of epistasis, even if assumed to be minimal in diploid model crop species.

In-depth GEA studies on the molecular mechanisms of evolutionary divergence involving the genomics and functional genetic dissection of adaptive loci enable addressing long-term transdisciplinary questions such as 1) how genomic features (i.e., meiotic crossover hot- vs. cold-spots, as pericentromeric regions and inversions) impact the rate of adaptation and modulate adaptive evolution to new environments (Huang et al., 2020; Huang and Rieseberg, 2020; Todesco et al., 2020), 2) how old adaptive haplotypes (standing variation) and more recent recruitment of novel mutations balance during the rapid events of changing climate (Jones et al., 2012a; Jones et al., 2012b), 3) what effects the noncoding *cis*-regulatory mutations contribute to the genomic basis of adaptation, and 4) to which scale epigenomic marks [i.e., chromatin accessibility, histone profiling, transposable elements, and sRNA (Kaasik and Chi Lee, 2004; Slotkin and Martienssen, 2007)] regulate plastic gene expression within the same genotype (Bossdorf et al., 2008), and may be transferred *via* transgenerational epigenetic inheritance (Heard and Martienssen, 2014; Boskovic and Rando, 2018; Hu et al., 2018; Lacal and Ventura, 2018), eventually impacting divergent adaptation in natural populations (Chinnusamy and Zhu, 2009).

Ultimately, GEA is empowering the understanding on how plant genomes interact with their environment while shaping adaptive phenotypes. Such mechanistic insights of the genome functionality in the wild promise leveraging the characterization of landraces and CWR to assist prebreeding efforts through multi-dimensional adaptive scores (*e.g.* GEAVs), as well as the identification of underlying factors that may facilitate or constrain future adaptive responses to changing climate.

Lastly, GEA studies offer new possibilities to efficiently unlock crop diversity for climate adaptation (Tanksley and McCouch, 1997). Unexplored variation already contained in genebanks (Smale and Jamora, 2020) may speed up resilience to extreme temperatures, and more frequent drought and flooding events (Dwivedi et al., 2016). The modern genome–environment framework, coupled with explicit ecophysiological indices and last-generation association models, promises a scalable strategy to assist with the identification and deployment of exotic variation capable of maturing earlier and harvesting acceptably in erratic climatic conditions. Levering reverse genomic and ecological resources for CWR and landraces will improve available pipelines such as focused identification of germplasm strategy, FIGS (Stenberg and Ortiz, 2021). Such efforts to prebreed resilient crop genotypes with greater accuracy may ultimately enable small-scale farmers’ adaptation to changing climate (Razzaq et al., 2021).

## Data Availability

The original contributions presented in the study are included in the article. Further inquiries can be directed to the corresponding author.
